# Current knowledge on the role of extracellular vesicles in endometrial receptivity

**DOI:** 10.1186/s40001-023-01459-y

**Published:** 2023-10-30

**Authors:** Cong Sui, Zhiqi Liao, Jian Bai, Dan Hu, Jing Yue, Shulin Yang

**Affiliations:** Reproductive Medicine Center, Tongji Hospital, Tongji Medical College, Huazhong University of Science and Technology, Jiefang Avenue 1095#, Wuhan, 430030 People’s Republic of China

**Keywords:** Endometrial receptivity, Embryo implantation, Extracellular vesicles, Non-invasive biomarker, Therapeutic mediator

## Abstract

Endometrial receptivity has been widely understood as the capacity of the endometrium to receive implantable embryos. The establishment of endometrial receptivity involves multiple biological processes including decidualization, tissue remodeling, angiogenesis, immune regulation, and oxidative metabolism. Extracellular vesicles (EVs) are lipid-bilayer-membrane nanosized vesicles mediating cell-to-cell communication. Recently, EVs and their cargo have been proven as functional factors in the establishment of endometrial receptivity. In this review, we comprehensively summarized the alteration of endometrium/embryo-derived EVs during the receptive phase and retrospected the current findings which revealed the pivotal role and potential mechanism of EVs to promote successful implantation. Furthermore, we highlight the potentiality and limitations of EVs being translated into clinical applications such as biomarkers of endometrial receptivity or reproductive therapeutic mediators, and point out the direction for further research.

## Background

The success of human embryo implantation relies on the synchronized dialogue between a receptive endometrium and a functional blastocyst [1]. This process, dependent on ovarian steroids, can only occur during a limited period from days 20 to 24 of the menstrual cycle named the ‘window of implantation’ (WOI) [1]. Perturbation of this process leads to implantation failure, accounting for approximately 75% of human pregnancy losses [2–7]. The endometrium plays a critical role in this process, as compromised factors within the endometrium account for two-thirds of implantation failures [1, 6, 8–10]. The endometrium not only provides a suitable microenvironment for early embryo development, but also actively modulates the process of implantation via intricate signaling networks [11–13].

Extracellular vesicles (EVs) have emerged as a potent mediator of signaling between the endometrium and embryo [11, 14–17]. They encapsulate diverse molecules for intercellular communication, including proteins, lipids, and RNAs [11, 18–22]. In the past decade, EVs have gained significant attention in the field of reproductive pathophysiology due to their diverse roles in gametogenesis and dynamic embryo–endometrial cross-talk [23, 24].

In this review, we retrospected the novel findings revealing the vital role and potential mechanism of EVs in the establishment of endometrial receptivity during embryo implantation. In addition, the utility and potential of EVs as clinical and therapeutic mediators, along with their limitations, were emphasized. This paper will contribute to a novel perspective on understanding the establishment of endometrial receptivity, thereby offering new insights for the treatment of reproductive system disorders.

## Extracellular vesicles

Extracellular vesicles are bilayer-membrane nanosized vesicles (30–1000 nm) secreted by cells as a part of their normal physiological processes and also during pathological conditions [25–27]. EVs are composed of three subtypes, including exosomes (50–150 nm), microvesicles (MVs) (100–1000 nm), and apoptotic bodies (500–5000 nm) [28–30]. They can be broadly classified into two categories: small EVs with a size range of approximately 30–150 nm and large EVs with a size range of approximately 150–500 nm [31–33]. The small EVs comprise exosomes of endocytic origin, which form via two times of membrane bubbling [28, 34–36]. And the large EVs include MVs that are produced by cell shedding directly [28, 34, 35, 37]. However, due to the challenge of complete separation between MVs and exosomes, it was thought difficult to discern the distinct functions of various types of EVs [28].

Exosomes have been investigated in 1908s [38]. However, it was after the discovery of their capability to facilitate intercellular transportation of functional mRNAs and microRNAs (miRNAs) that EVs started garnering increased attention from researchers [39, 40]. To date, EVs are known transporters of a variety of molecules, such as nucleic acids, proteins, and lipids to conduct intercellular communication [39–41]. The molecules in the EVs remain stable due to being protected from enzyme degradation [28]. EVs can interact with certain target cells specifically due to carrying the surface receptors or ligands of original cells [35]. The way EVs transmit signals between cells provides a new mechanism for intercellular communication in addition to contact-dependent and autocrine, paracrine, or endocrine signals [28]. EVs have been demonstrated to be secreted by the uterus and embryo, playing a crucial functional role in embryo–endometrial dynamic communication [14, 15, 27, 42–49]. Therefore, it is important to explore the role and mechanism of EVs in endometrial receptivity during embryo implantation.

## Changes in endometrium-derived EVs during the endometrial receptivity phase

Cyclic changes in the endometrium are regulated by estrogen and progesterone [12, 50]. During the secretory phase of the human menstrual cycle, estrogen and progesterone induce decidualization of human endometrial stroma, resulting in a receptive decidua that is not dependent on implantation [51, 52]. Decidualization refers to the process of endometrial stromal cells (ESCs) undergoing epithelioid transformation during embryo implantation [53, 54]. Adequate decidualization plays a crucial role in ensuring successful pregnancy establishment, regulating trophoblast invasion, and optimizing placental perfusion [52]. EVs have been isolated from cultured ESCs, as well as decidualized stromal cells [15, 55–57]. Ma, Q. et al. discovered that during decidualization, primary human endometrial stromal cells (hESCs) were found to secrete EVs, which is controlled by a conserved HIF2α-RAB27B pathway [58]. Their study also demonstrated that the internalization of EVs carrying the glucose transporter 1 (GLUT1) by hESCs, promotes glucose absorption, thereby supporting and advancing the decidualization process [58]. Gurung et al. indicated hESCs response even before decidualization, and EVs of poor decidualized stromal cells are significantly different from those that readily decidualize [59]. EV-proteins from poorly decidualized ESCs may be detrimental to the core functions of endometrial receptivity, placentation, menstrual health, and endometrial regeneration via dysregulated pathways including complement and coagulation cascades, innate immune response, B cell receptor signaling and platelet degranulation [59].

By utilizing estrogen and progesterone to mimic the menstrual cycle phases in vitro cultured RL95-2 cells, Hart, et al. demonstrated that while endometrial-derived EVs were secreted independently of hormonal stimulation, their sizes were significantly altered by it [60]. Proteomics analysis revealed that EVs in the receptive phase group induced by estrogen and progesterone are implicated in various processes, including endometrial receptivity (ACE2, PDIA3, PLAT, SLC6A6, TSPAN6, DNAJB1, LUC7L3, and INHBB), embryo development (FUCA1 and LDHA), and embryo implantation (CDH5, HSPG2, KIF5C, EIF4E, FSTL1, ITGA2B, and AASDHPPT) [46, 49, 60–63]. In the secretory (estrogen plus progesterone-driven) versus proliferative (estrogen-driven) phases of fertile women, Rai et al. found an enrichment of invasion-related proteins (LGALS1/3, S100A4/11), proving that EVs from estrogen plus progesterone-driven versus estrogen-driven human endometrial epithelial cells (EECs) promote trophectoderm cell invasion [64]. EVs derived from an original endometrial epithelial cell line treated with estrogen and progesterone exhibited a rapid and significant increase in the adhesive and invasion capacity of HTR8 cells by promoting outgrowth on fibronectin [46]. This finding is consistent with the results obtained from their proteomic analysis, which has shown selective enrichment in secretory EVs of the cell surface (HSPG2, CD55, CD47, EGFR), and secreted (CYR61) molecules, cytoskeletal regulators (CLDN3, CELSR2, PARVA), enzymes (ADAMTS15, DPP3, ANPEP, ADAM10) [46]. Fatmous et al. detailed that estrogen/progesterone-regulated endometrial EVs (but not estrogen alone-regulated EVs) promote human trophectodermal cell invasion via MAPK activation and that pharmacological inhibition of MAPK activation abrogates this process [65]. Therefore, it is crucial to understand the role of EVs from the endometrium in regulating endometrial receptivity.

## The role of endometrium-derived EVs in endometrial receptivity

**Fig. 1:**
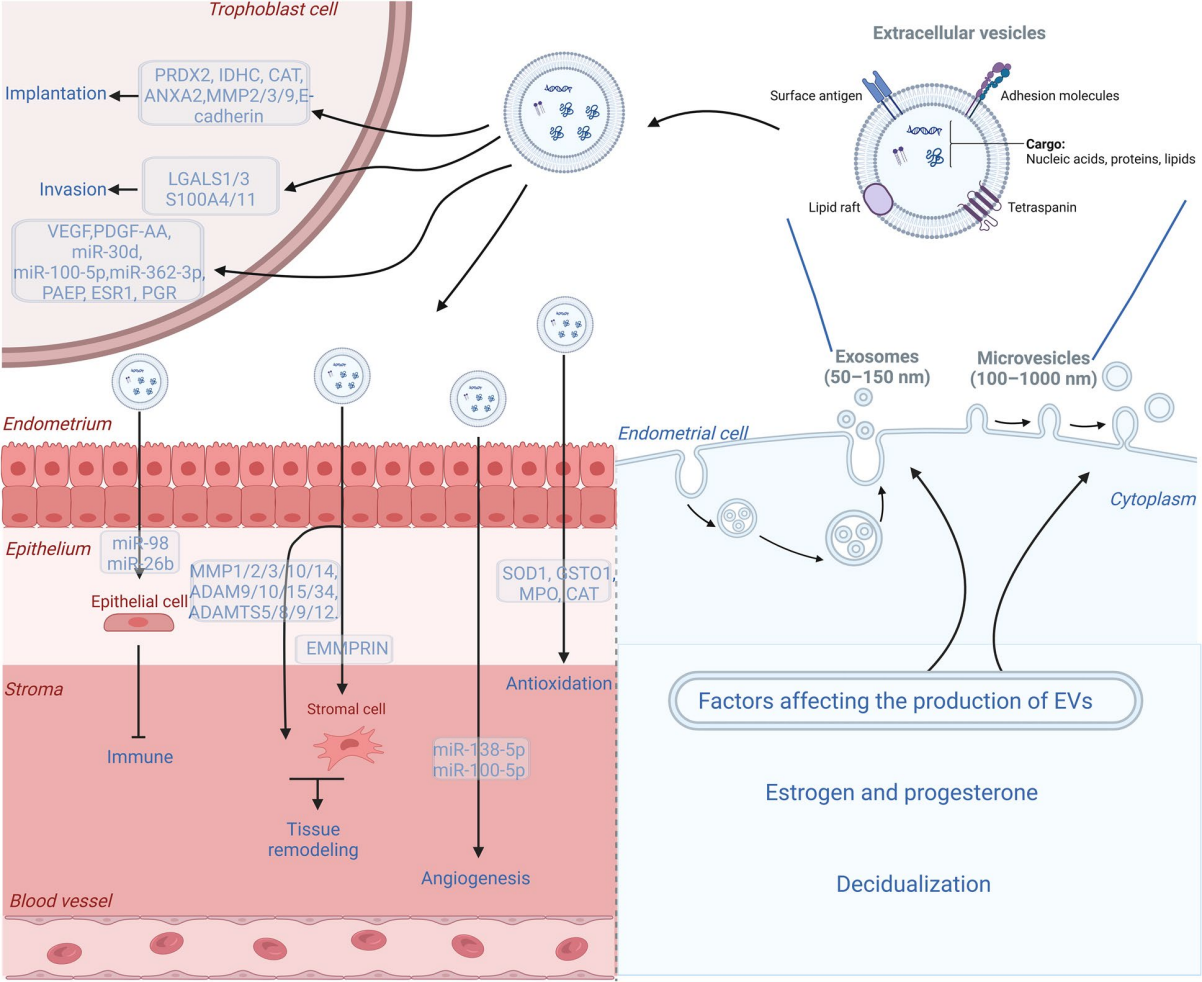
The role of endometrial cell-derived EVs in regulating the endometrial receptivity and the trophoblast function. The EVs can regulate tissue remodeling, promote angiogenesis, exert antioxidant activity, and exert immunosuppressive function. Moreover, the EVs promote the migration and invasion of the trophoblast cells

Endometrial receptivity refers to the ability of the endometrium to facilitate normal implantation, and optimal receptivity is crucial for successful implantation processes that establish a healthy pregnancy [66–68]. During the mid-secretory phase of the menstrual cycle, the human endometrium undergoes a brief period of receptivity characterized by its ability to provide an immune-privileged and nutritive environment for the embryo, which is called WOI [69, 70]. EVs can be isolated from endometrial cells and have been shown to exist in uterine fluid [20, 46, 64, 71, 72]. EVs from the uterine fluid recapitulate the dynamic physiological state depending on the different phases of the menstrual cycle [20, 46, 64, 71–74]. Here, our focus lies in exploring the functional role of endometrium-derived EVs in various aspects of endometrial receptivity, which is presented in Fig. 1.

### The role of endometrium-derived EVs in tissue remodeling

The uterus is a unique organ that experiences significant tissue remodeling throughout each menstrual cycle, pregnancy, and postpartum period [75]. The metzincin gene superfamily can be found in membrane-anchored or soluble forms and plays pivotal roles in inflammation, tissue, and extracellular matrix remodeling, as well as organogenesis [76, 77]. During the endometrial remodeling in the menstrual cycle, several matrix metalloproteinases (MMPs) are highly expressed in the endometrium at the initiation of menstruation, then positively regulated by estrogen and suppressed by progesterone [46, 55, 78–81]. At the time of embryonic implantation and endometrial decidualization, the process of endometrial remodeling is also precisely regulated by the expression of MMPs and their inhibitors (Tissue Inhibitors of Metalloproteinase Inhibitors, TIMPs) [82, 83]. To date, the proteins MMP-1, -3, and -10, A disintegrin and metalloproteinase (ADAM) -9, -10, -15, and -34, as well as A disintegrin and metalloproteinase with thrombospondin motifs (ADAMTS) -5, -8, -9, and -12 have been observed in stromal cell-derived EVs [39, 84, 85]. A potential mechanism of action for endometrium-derived exosomal MMPs involves the activation and degradation of other proteins [86]. For instance, human endometrial-derived EVs have been demonstrated to contain MMPs that are internalized by human uterine fibroblasts and promote the production of MMP-1/-2/-3, which are critical factors for tissue remodeling [75]. Moreover, the extracellular matrix metalloproteinase inducer (EMMPRIN), a transmembrane glycoprotein belonging to the MMP family, plays a pivotal role in embryo implantation and placentation by stimulating the expression of MMPs in uterine stromal cells through microvesicle shedding, particularly MMP-2 and -14 [81, 87, 88]. The MMP-2 is a prominent EV cargo protein of decidualized mouse endometrial stromal cells (mESCs), which modulates uterine remodeling during decidualization [89]. The MMP-14 is expressed at the fetal–maternal interface in both human and mouse models, with pronounced upregulation observed in extravillous trophoblast cells, which plays a pivotal role in trophoblast invasion and influences the outcome of pregnancy [90–94]. Consequently, exosomal active MMPs can modulate the activity and bioavailability of various factors, thereby influencing the exosome-mediated communication between the embryo and endometrium.

### The role of endometrium-derived EVs in angiogenesis

The endometrium is a cyclic dynamic tissue with significant physiological angiogenesis occurs. In a menstrual cycle, the arterioles are straight in the proliferative phase and become spiraled and transformed into a low-resistance vascular network by dilation, and disorganization of the vascular smooth muscle cells in the secretory phase [95–97]. Endometrial receptivity and successful embryo implantation require coordinated development and maintenance of blood vessels at the maternal–embryonic interface to provide a nutritional environment [98–100]. A significant angiogenic proliferation occurs concomitantly with the process of uterine decidualization. EVs serve as a mechanism of intercellular communication that exerts significant influence on various endothelial functions, such as vascular tone regulation, the interaction between endothelial cells and smooth muscle cells or pericytes, and angiogenesis [101]. Ma, Q. et al. reported that EVs secreted by decidualized mESCs augmented the differentiation potential of mESCs and stimulated their production of angiopoietin 2 [89]. Additionally, EVs derived from ESCs can stimulate the proliferation of human endothelial cells and enhance vascular network formation [58]. Stromal cell-derived EVs also can induce tubercle vein endothelial cells in vitro, indicating their potential role in regulating angiogenesis during implantation [58]. Endometrial cell-derived EVs-associated microRNA-138-5p (by adjusting angiogenic player GPR124) and miR-100-5p enhance angiogenesis during the implantation process [102, 103]. There is evidence suggesting that endometrial mesenchymal stromal cells (endMSCs) exert a paracrine influence on embryonic activities, thereby facilitating endometrial angiogenesis and vascularization through the release of EVs [104]. When co-cultured with murine embryos, EVs derived from endMSCs enhance blastocyst cell proliferation and expansion rate, while also inducing the release of pro-angiogenic factors such as vascular endothelial growth factor (VEGF) and platelet-derived growth factor-AA (PDGF-AA) from the embryos [105].

### The role of endometrium-derived EVs in immune regulation

The homeostasis between active immunity and tolerance at the maternal–fetal surface between the uterus and embryo is crucial for a successful pregnancy [106, 107]. The embryos can secrete immunosuppressive IL-10, hCG, and HLA-G, protecting themselves from maternal immune attacks [15, 107, 108]. Decidual stromal cells may play an essential role during the pregnancy by inhibiting T cell function and promoting regulatory T cells (Tregs) through the activation of indoleamine-2,3-dioxygenase (IDO), prostaglandin E2, programmed death ligand (PD-L)1, and interferon-gamma (IFN-γ) [109–111]. Recently, uterine fluid EVs were proven to possess potent immunomodulatory effects on the maternal immune system during implantation. Nakamura et al., reported that uterine fluid-EVs via bta-miR-98 collaborate with bta-miR-26b negatively regulated several immune system-related genes (CTSC, IL6, CASP4, IKBKE, and PSMC6, CD40, and IER3, respectively) in bovine EECs during receptivity phase [44, 112]. By global analysis of differentially expressed proteins between EVs, revealed EVs affected the down-regulation of “neutrophil activation involved in immune response” and “neutrophil-mediated immunity” [112].

### The role of endometrium-derived EVs in antioxidant activity

Oxidative metabolism is the main source of energy in humans. There is a defense system against reactive oxygen species (ROS) to maintain a balance between pro-oxidants and antioxidants [113]. Physiological levels of ROS play a crucial regulatory role through diverse signaling pathways in folliculogenesis, oocyte maturation, endometrial cycle, luteolysis, implantation, embryogenesis, and pregnancy [114]. Mammalian blastocysts are hatched from their zona pellucida before implantation. Thomas, M. et al. have demonstrated that peri-hatching blastocysts generate a significantly high level of ROS for an extremely brief period in comparison to pre-hatching (unhatched) and post-hatching (hatched) blastocysts, due to a decline in the antioxidative superoxide dismutase (SOD) activity and an outburst of superoxide anion radical generation in the peri-hatching (peri-implantation) blastocysts of mice [115]. However, embryos at this stage are particularly susceptible to oxidative stress and damage. The antioxidants protect embryos from ROS-mediated damage, implantation failure, and pregnancy loss [116, 117]. There are already several studies indicating that uterine lavage contains a variety of antioxidants that protect pre-implantation embryos by reducing oxidative damage [118–122]. Rai et al. used mass spectrometry-based quantitative proteomics to show that compared to infertile women, EVs isolated from uterine lavage of fertile women in the secretory phase are enriched with proteins that have been implicated in antioxidant activity, including SOD1, GSTO1, MPO, and CAT [64].

### The role of endometrium-derived EVs in trophoblast adhesion and invasion

The blastocyst attaches to the receptive endometrium through the processes of adhesion and invasion [28]. It has been proved that EVs secreted by endometrial cells promote these processes [46, 73–75]. When compared to the proliferative phase, proteomic studies of EVs from the uterine fluid of fertile and infertile women revealed an enrichment of proteins linked to invasion (LGALS1/3, S100A4/11) and implantation (PRDX2, IDHC, CAT, ANXA2) in secretory phase [64]. Decidual stromal cell-derived EVs can also be internalized by trophoblast cells, thereby inducing invasion through the SMAD2/3-N-cadherin signaling pathway [57]. EVs derived from human endMSCs have been observed to significantly promote blastomere division, augment the total cell number of mice embryos, and embryo hatching of pre-implantation mice embryos [105]. The proteomic analyses of the EVs derived from human endMSCs found proteins related to embryo development (transferrin, vinculin, and fibronectin) and implantation (MMP-2, -3, and -9, and E-cadherin) [105]. Gurung et al. used EECs-derived EVs to intervene in human trophectodermal spheroids and came to a similar result [74]. Transcriptomic suggests miRNAs of endometrial EVs (e.g., hsa-miR-30d, miR-100-5p, hsa-miR-362-3p) and mRNAs (PAEP, ESR1, PGR) can reprogram gene expression of trophoblast cells [21, 49, 71, 103]. It is worth noting that the enhanced adhesion can be partially reduced by EV uptake inhibitors, providing evidence for the impact of endometrium-derived EVs [73]. The findings of our research team reveal an intriguing observation that EVs derived from women experiencing recurrent implantation failure (RIF) exhibit a suppressive effect on the growth and invasion of embryos [11]. The subsequent experiment demonstrated that EVs of RIF patients inhibit the proliferation, migration, and invasion of HTR8/SVneo cells [17].

Taken together, endometrium-derived EVs play crucial roles in decidualization, tissue remodeling, angiogenesis, immune regulation, and oxidative metabolism during the establishment of endometrial receptivity. The significance of the EVs secreted by the embryo in mediating endometrial receptivity should also be emphasized.

## The embryo-derived EVs modulate endometrial receptivity

Studies have demonstrated that the endometrial receptive stage undergoes significant changes during the pre-implantation phase [123–129]. Embryo-derived factors like human chorionic gonadotropin (hCG) and interleukin-1beta (IL-1β) are thought to mediate the modulation of the receptive endometrium [128, 130]. Indeed, embryos can also produce EVs, and the production rate and type of EVs are subject to change during their development process [14, 15, 24, 131–135]. The culture medium for both day 3 (D3) and day 5 (D5) in vitro embryos contains EVs ranging from 50 to 200 nm, with an average size of 100 nm [53]. Both D3 and D5 culture media of human embryonic EVs are positive for CD9, CD63, ALIX, and HLA-G, which are enriched with mRNAs encoding pluripotency genes including Oct4, Sox2, Klf4, c-Myc, and Nanog [136–140]. The embryo-derived EVs can traverse the zona pellucida, and exert both autocrine effects on trophoblast cells and paracrine effects on the endometrium [15, 135, 141]. The evidence has demonstrated that embryos release a diverse population of EVs containing embryonic-specific molecules, which are selectively targeted to both epithelial and stromal cells. This novel mechanism of intercellular communication facilitates cellular activities such as adhesion and migration, implying the potential for modifying the endometrial genome during embryonic development. [15, 53, 135, 138, 142, 143]. Interestingly, Es-Haghi et al. discovered that only embryos with a favorable prognosis exhibited the observed effects, while degenerated embryos failed to elicit any alterations [42]. The proposed hypothesis suggests that the signaling from the embryo to the endometrium serves as a component of a quality control mechanism employed for evaluating the developmental competence or incompetence of the embryo [52, 144]. Furthermore, Nakamura et al. assessed the potential role of EVs containing interferon tau (IFNT) on primary uterine EECs. The EVs secreted by the blastocyst after hatching from the zona pellucida regulate genes and maintain progesterone production for the successful establishment of pregnancy [47].

**Fig. 2:**
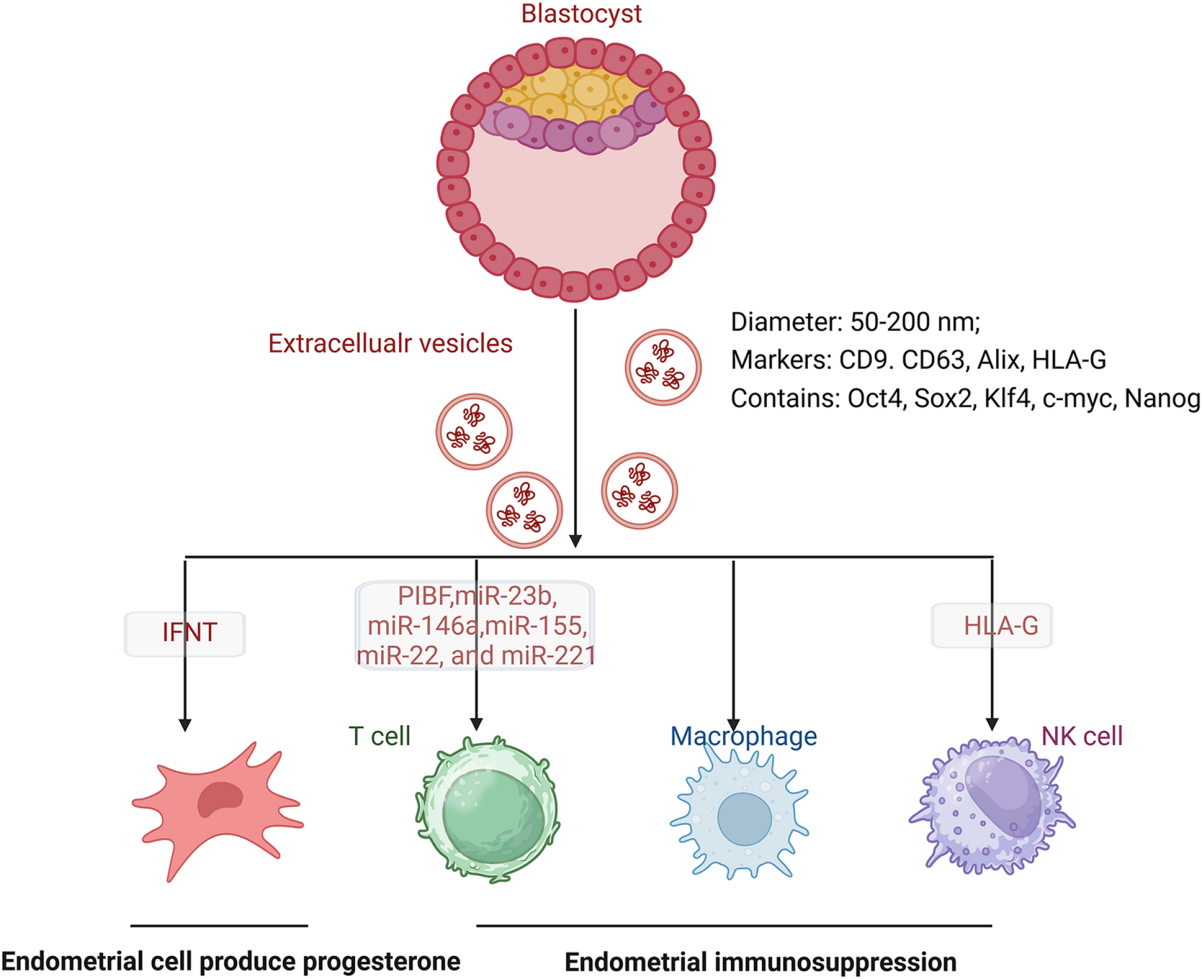
The embryo-derived EVs regulate the endometrial receptivity via regulating progesterone production and immunosuppression

Owing to the coordinated efforts of monocytes, Tregs, natural killer (NK) cells, and a balanced cytokine profile, the developing embryo can thrive in an immunologically favorable environment [145]. Embryos also secrete immunosuppressive molecules in EVs and stimulate the production of immunosuppressive factors to evade maternal immune responses. Trophoblast-derived EVs dose-dependently enhanced monocyte migration and significantly upregulated the production of IL-1β, IL-6, Serpin-E1, granulocyte colony-stimulating factor, granulocyte/monocyte colony-stimulating factor, and tumor necrosis factor-alpha [146]. Likewise, the trophoblast-derived EV-associated HSPE1 and miRNA cargo, including hsa-miR-23b, hsa-miR-146a, hsa-miR-155, hsa-miR-22, and hsa-miR-221, play a crucial role in the differentiation of Tregs at the feto-maternal interface [147]. The mouse embryonic EVs containing progesterone-induced-blocking factor 1 (PIBF), which interact with CD4+ and CD8+ peripheral T cells and stimulate IL-10 production, have been suggested to regulate NK cell activity [145]. Moreover, the trophoblast can express histocompatibility antigen, class I, G (HLA-G), which necessitates intercellular transport via EVs and serves as a defense mechanism against NK cell-mediated death [15, 148]. The mechanism by which embryo-derived EVs regulate endometrial receptivity is shown in Fig. 2.

In summary, embryonic-derived EVs possess the potential to modulate endometrial responses, including enhancing progesterone production and stimulating immunosuppressive factors. This contributes to establishing endometrial receptivity and facilitating successful implantation.

## The possible clinical utility of EVs in embryo implantation

### EVs as biomarkers of endometrial receptivity

The ability to accurately detect endometrial WOI would significantly enhance the success rates of fertility treatments [149, 150]. Though limited in value, we used ultrasound (endometrial thickness, character, volume, and blood flow patterns), histological (pinopods), biochemical (integrins, leukemia inhibitory factor, homeobox A10, mucin 1, calcitonin, cadherin 6, and cyclo‑oxygenase 2) markers to assess endometrial receptivity for a long time [1, 13, 61, 151–158]. Transcriptomics (i.e., endometrial receptivity array, ERA) is currently regarded as the most established technology available for assessing the endometrial factor [152, 156, 159–162]. However, the use of transcriptomics has not demonstrated improved pregnancy outcomes in patients with RIF [12, 163–168]. There is still a challenge in diagnosing endometrial receptivity due to the absence of an accurate, noninvasive, and clinically applicable test.

The ERA of endometrial tissue revealed WOI displacement in 25.9% of patients with RIF and 12% of the control population [169]. Referencing the ERA gene list, the transcriptome of EVs from uterine fluid correlates with the endometrial tissue transcriptome [20]. Moreover, the proteome of uterine fluid-derived EVs highlights a distinct protein landscape in EVs between fertile and infertile women to predict WOI [64]. Furthermore, other investigations have explored the potential of EVs from the uterine fluid as predictors of receptivity. Li et al., have identified EVs from uterine fluid containing small non-coding RNA biomarkers (11 miRNAs and 1 piwi-interacting RNA) of endometrial receptivity and implantation success [21]. Ibañez-Perez, J. et al., introduced protocols to analyze the miRNAs in EVs from uterine fluid and used hsa-miR-99b-5p (employing the PBP-N detection method) to predict the endometrial receptivity [170]. The proteome also highlights the EVs from the uterine fluid as potential applicability for biomarkers in endometrial receptivity. Rai et al. showed that EVs from the uterine fluid of fertile women carry known receptivity protein markers (S100A4, FGB, SERPING1, CLU, ANXA2) [64]. Marina Segura‑Benítez et al. investigated and identified 82 proteins in EVs secreted by primary human EECs collected from fertile women and cultured in vitro could define them as novel biomarkers of endometrial receptivity and implantation success [171]. Gurung et al. provides insight into EVs-proteomes as a benchmark of well-decidualized endometrial stromal cell, which may be beneficial to the functions of endometrial receptivity [59]. In the field of assisted reproduction, uterine fluid-derived EVs may serve as a less-invasive molecular marker to accurately determine the optimal timing for embryo transfer [71]. Therefore, transcriptomic and proteomic analysis of human endometrium-derived EVs could make it possible to use it as a less invasive way to detect endometrial receptivity. However, it is important to acknowledge that inadequate isolation and purification of EVs may compromise the validity of results, thereby confounding data interpretation [24].

### EVs as reproductive therapeutic mediators

EVs are enriched in RNA transcripts and protein molecules, which are crucial for implantation [46, 64, 73, 170]. The study of endometrial EVs in patients with RIF confirmed the negative effect of EVs on endometrial receptivity and embryo implantation [11, 17]. This has led to the interest in harnessing EVs for therapeutic development. Marinaro et al. indicated that human EVs of endometrial stem cell origin could improve the developmental competence of aged oocytes and increase the odds of implantation and subsequent delivery [172]. Hamed Hajipour et al. used uterine fluid-derived EVs as a drug carrier system to deliver the hCG to the endometrial cells. The EV-encapsulation enabled a steady release of hCG over a period of 72 h, resulting in a significant increase in the effect of hCG on the expression of LIF and Muc-16 [173]. Morteza Taravat et al. loaded rosmarinic acid into serum-derived exosomes. The EV-encapsulated rosmarinic acid exerts an anti-inflammatory effect by inhibiting the TLR4–NLRP3 signaling pathway, thereby ameliorating pathological changes, and reducing myeloperoxidase production in a murine model of endometritis [174]. Therefore, natural, or engineered EVs hold potential as therapeutic agents for reproductive disorders in the future. Compared to conventional drugs, therapeutic EVs offer numerous advantages. Owing to the lipid membrane, EVs are more easily taken up by cells to exert a therapeutic function. Moreover, EVs can also be engineered to express surface ligands to target specific recipient cell types, which promotes the efficiency of the use of EVs [175].

The considerable attention garnered by applications of natural, or engineered EVs with predetermined contents for therapeutic purposes. However, some challenges in the clinic’s use of EVs still need to be addressed: (1) there is a lack of universally recognized standards for the separation, concentration, as well as nomenclature for subclassifying EVs based on their diverse biophysical properties [29]. (2) Current technologies relying on ultracentrifugation, ultrafiltration, antibody-coupled magnetic beads, and cryoelectron microscopy encounter significant challenges in terms of exosome separation, purification, and quantification [176]. (3) The heterogeneity of EV preparations will lead to variations in yields and concentrations, posing challenges for their clinical application [32]. Besides, the heterogeneity of EV contents will introduce ambiguity to the underlying mechanism of EV treatment. (4) The safety issues of EVs and engineered EVs should be considered and assessed by long-term monitoring [29].

## Conclusions

EVs play a crucial role as bidirectional signaling regulators in embryo implantation at the interface between the embryo and maternal tissues. Nevertheless, the exact mechanisms underlying the embryo–endometrial cross-talk mediated by EVs are not fully comprehended and additional research is necessary. In this paper, we present a comprehensive review of the studies that support the involvement of EVs in the intricate process of endometrial receptivity and their pivotal role in embryo-mediated modulation of the receptive endometrium (Table 1). Although we acknowledge the heterogeneity of endometrial EVs in terms of size, current data only provide pooled estimates for this diverse population. Further research is necessary to comprehend the unique biological impacts of diverse cargos carried by EVs of varying sizes. In addition, our understanding of the roles of endometrial EVs is currently limited to their protein and RNA cargo. The development of omics technologies will undoubtedly enhance our comprehension of the pivotal role played by EVs in endometrial receptivity. Future research should prioritize the development of techniques for isolating and characterizing endometrial EVs. This will pave the way for the development of noninvasive biomarkers for endometrial receptivity and therapeutic mediators for pathophysiology.

**Table 1:** The characteristics of the endometrium- and embryo-derived EVs

Source	Markers	Contents	Function	References
Endometrial epithelial cells	Alix, HSP70 TSG101, CD9, CD63	1: Proteins: SOD1, PRDX6, PRDX1, TMP4, PARK7 2: Protein: EMMPRIN 3: Proteins: BMPR2, DDR1, IGSF8, MST1R4 4: Proteins: COPS3, CUL3, NOTCH1, PLCG1, ADAM10 5: Proteins: CSTB, DDR1, RAB25, ST14, TXN 6: sncRNAs: miR-100-5p	1: Enhance trophectoderm invasion 2: Stimulates metalloproteinase production 3: Epithelial cell migration 4: Embryo development 5: Cell invasion 6: Promote trophoblast migration and invasion and promote angiogenesis	[65] [75] [103] [65]
Endometrial stromal cells	CD63 CD81	1: sncRNAs: miR-138-5p, miR-100-5p 2: Proteins: MMP-1/3/10, ADAM9/10/15/34, ADAMTS5/8/9/12	1: Induce tubercle vein endothelial cells and angiogenesis 2: Regulates embryo implantation and early pregnancy 3: Induce the release of VEGF and PDGF-AA of embryo	[102] [58] [104]
Uterine fluid	ALIX, CD63, TSG101, CD9	*1: RNAs:* AC114491.1, *AC008608.2*, *PMS2P5*, *C10orf99*, *NPTN-IT1*, *AC012358.3*, *ANKRD18A*, *GLIS2-AS1*, AC011447.7, AL009174.1, *C1QTNF2*, *TMED6*, *AC016355.1, AL021392.1* 2: RNAs: *CD200R1*, *FAM66B*, *AL391834.1*, *WNT9B, CEC*R7 3: Proteins: MPO,PRDX1/2, TXN, PARK7 4: Proteins: LGALS1, LGALS3, VIM 5: sncRNAs: hsa-miR-501-5P, hsa-miR-411-3P, hsa-miR-18a-5P, hsa-miR-196a-5P, hsa-miR-493-5P, hsa-miR-497-5P	1: Selectively detected in women with successful implantation 2: Selectively detected in women with failed implantation 3: Regulate antioxidant activity 4: Invasion-related proteins 5: TGF-β receptor signaling pathway, Hippo signaling pathway, and immune response	[20] [65] [21]
Trophoblast	PD-L1, CD63 CD81 CD9 PLAP MIC-A/B ULBP1-5	*1: RNAs:* *HSPE1* 2: sncRNAs: has-miR-23b 3: sncRNAs: hsa-miR-146a, hsa-miR-155	1: Regulate T_reg_ cells 2: Inhibit the Th17 signaling 3: Regulate T_reg_ cells	[147] [148]
Embryo	CD9 CD63 Alix HLA-G	1: RNAs: *Oct4, Sox2, Klf4, c-myc, Nanog* 2: Proteins: IFNT, HLA-G, PIBF	1: Regulate the production of progesterone 2: Modulate the activity of decidual NK cells, macrophages, T cells, and B cells	[48] [136–140] [148]

## Data Availability

All data generated or analyzed during this study are included in this published article.

## Abbreviations

EVs: Extracellular vesicles
WOI: Window of implantation
MVs: Microvesicles
miRNAs: MicroRNAs
ESCs: Endometrial stromal cells
hESCs: Human endometrial stromal cells
GLUT1: Glucose transporter 1
EECs: Endometrial epithelial cells
MMPs: Matrix metalloproteinases
TIMPs: Tissue Inhibitors of Metalloproteinase Inhibitors
ADAM: A disintegrin and metalloproteinase
ADAMTS: A disintegrin and metalloproteinase with thrombospondin motifs
EMMPRIN: Extracellular matrix metalloproteinase inducer
mESCs: Mouse endometrial stromal cells
endMSCs: Endometrial mesenchymal stromal cells
VEGF: Vascular endothelial growth factor
PDGF-AA: Platelet-derived growth factor-AA
Tregs: Regulatory T cells
IDO: Indoleamine-2,3-dioxygenase
PD-L: Programmed death ligand
IFN-γ: Interferon-gamma
ROS: Reactive oxygen species
SOD: Superoxide dismutase
RIF: Recurrent implantation failure
hCG: Human chorionic gonadotropin
IL-1β: Interleukin-1beta
D3: Day 3
D5: Day 5
IFNT: Interferon tau
NK: Natural killer
PIBF: Progesterone-induced-blocking factor
HLA-G: Histocompatibility antigen, class I, G
